# The pros and cons of researching events ethnographically

**DOI:** 10.1177/1466138117709293

**Published:** 2017-05-16

**Authors:** Thijs van Dooremalen

**Affiliations:** University of Amsterdam, The Netherlands; Lotte Meinert and Bruce Kapferer (eds), *In the Event – Toward an Anthropology of Generic Moments.* New York: Berghann Books, 2015; 180 pp. Pnina Werbner, Martin Webb and Kathryn Spellman-Poots (eds), *The Political Aesthetics of Global Protest: The Arab Spring and Beyond.* Edinburgh: Edinburgh University Press, 2014; 410 pp. Michael A. Gould-Wartofsky, *The Occupiers: The Making of the 99 Percent Movement.* Oxford: Oxford University Press, 2015; 316 pp.

**Keywords:** events, social change, ethnography, fieldwork, protest

## Abstract

Events (remarkable, disruptive happenings) are important subjects of study for understanding processes of change. In this essay, I reflect upon the issue of what the ethnographic method has to offer for the analysis of this social phenomenon. To do so, I review three recently published ethnographic studies of events. My conclusion is that it is indeed a very useful method for understanding the feelings and ideas of people who are experiencing eventful situations, for instance around protests or natural disasters. However, using this method also brings about practical difficulties, such as the ‘luck’ that an event occurs at the ethnographic fieldwork site. Next, as transformative responses to events are not bound by the place or time of the happening, other methods (interviews, discourse analysis, surveys) that make it easier to follow them in varying locations and periods might be more suitable for getting a comprehensive picture of their meaning-making dynamics.

Social life is generally full of continuity. Every day, people encounter many happenings: they wake up, go to work, take care of their children, read or watch the news, go out, etc. Most of these activities are ordinary occasions that result in little or no change. Happenings that actually do result in substantial change are the exception (cf. Sewell, 2005: 226–8). Those are the kinds of happenings that scholars such as Sahlins (1985), Sewell (2005) and Berezin (2012) have referred to as ‘events’ (and that others have called ‘critical events’ (Das, 1995) or ‘critical moments’ (Bourdieu, 1988: 159–93)): occasions that surprise, shock, or excite people so much that they see them as inevitable signs that social reality should be transformed.

Therefore, if we want to understand processes of social change, it is important to solve event-related research quandaries. For example: under what circumstances do events come about? In what ways is the behaviour of people experiencing them different from that seen during more ‘settled times’ (Swidler, 1986)? To what extent have their lives – and society at large – been transformed once the event is over?

From a methodological point of view, the question is then: how can we best study them? At first glance, the ethnographic method seems to be perfectly suited to this task. An important characteristic of events is that they are indeterminate periods (Sahlins, 1985; Sewell, 2005; Wagner-Pacific, 2017). People who experience them go through ‘breaks with normal life’ (Sewell, 2005: 226–8); they have feelings and ideas that they would normally not have. Is the ethnographic method – the social scientific research method that probably gets closest to observing the intensities of people’s everyday lives (Jerolmack and Khan, 2014) – not particularly well-suited for investigating such periods?

In this review essay, I reflect on the pros and cons of studying events ethnographically. To do so, I examine three ethnographic studies that have been published recently, in which events play a significant role: *In the Event – Toward an Anthropology of Generic Moments* (Meinert and Kapferer, 2015), a collection of ethnographies of a wide variety of events that have happened all over the world; *The Political Aesthetics of Global Protest: The Arab Spring and Beyond* (Werbner, Webb and Spellman-Poots, 2014), an overview of the protest movements that have taken shape across the globe in the aftermath of the Arab Spring; and *The Occupiers: The Making of the 99 Percent Movement* (Gould-Wartofsky, 2015), a participant observational study of the (American) Occupy movement. These three books do not ostensibly present an overall picture of ethnographic event research. However, the variety in their respective theoretical perspectives and subjects of study gives an interesting overview of what this method has to offer with regards to the analysis of events. My central review questions are: *in which ways/to what extent can ethnography be used to study events? And, what types of social scientific insights can the ethnographic study of events generate?*

The three books differ in the extent to which they focus on events. As can be derived from its title, *In the Event – Toward an Anthropology of Generic Moments* (2015) centres on them. The main purpose of the book is to make event research more dominant within the field of anthropology. In the introductory chapter, Bruce Kapferer, one of the editors, claims that although anthropologists have often investigated events, their aim has always been to use them as lenses through which society at large could be studied – events as micro-representations of what is going on in society as a whole. *In the Event* aims to break with this tradition by observing events as autonomous realities: situations that not only exemplify general societal processes, but which by themselves can instigate social transformations. As Kapferer explains at the beginning of the chapter (p. 2):

Ultimately, the aim is toward the exploration of the event as a singularity of forces in which critical dimensions of socio-cultural existence reveal new potentials of the ongoing formation of socio-cultural realities. The approach to the event discussed here is one that goes beyond conventional perspectives of the event as representational of the social or of society and, instead, as a moment or moments of immanence and the affirmation and realization of potential.

This approach is inspired by Max Gluckmann’s anthropological Manchester School, which put ‘situational analysis’ at the core of its studies.

Each chapter in the book consists of an ethnographic description of a separate event. These happenings have taken place within different fields, at varying locations across the globe. The book covers, among other subjects, the satiric ‘re-enactment’ of a funeral during student elections at a South African university (Chapter 2), a value conflict within a Danish company (Chapter 7), and restructuring processes after a natural disaster in Mozambique (Chapter 9). Every chapter is written by scholars who had already done fieldwork at these sites. They did not specifically travel to them to study the respective events. The happenings occurred serendipitously during their fieldwork. This reveals a basic methodological problem for studying events ethnographically: one has to be at the fieldwork site during or shortly after the occasion. This means that to be able to make an event analysis, the researcher should already reside at or otherwise frequent the place of the happening.

Furthermore, she also needs to have substantial social scientific knowledge of the fieldwork site as well as the social capital that is necessary for properly conducting the research. Finally, the researcher has to be willing and able to give up initial fieldwork plans to devote attention to studying the event. Events happen more or less unexpectedly – this is one of the main reasons they can be so shocking (cf. Wagner-Pacifici, 2017). Thus, the researcher can never predict when and where an event will take place and how long it will last. In sum, this means that he must have some ‘luck’ to confront an event at the fieldwork site along with the practical possibilities (time, money) to start studying it.

The various analyses of *In the Event* do not employ one central definition of events. On the one hand, this is problematic, as it does not offer the possibility for building a general theory of the anthropology of generic moments (this is probably the reason there is no concluding chapter at the end of the book). On the other hand, this exposes the reader to many different approaches for the ethnographic study of events. One highly interesting approach is presented in Chapter 8, in which Stine Krøijer describes an episode of her fieldwork among radical left-wing activists in Northern Europe. During the 60th anniversary summit of NATO, held in Strasbourg in 2009, protesters congregated, resulting in a confrontation with police. They were crowded onto a bridge with nowhere to go and seemed destined to lose the conflict. However, because they formed a coherent group, with all the individuals staying close to one another and adopting the same physical pose, they were able to transform themselves from a multitude of bodies into one collective body. It proved so powerful that they were able to triumph in the confrontation and withdraw from the bridge, even though the police officers were better armed. This indicates that under specific circumstances, even a relatively small group of people can become very powerful, through creating a situation of collective effervescence (an event).

Another insightful perspective is offered in Chapter 5, which shows how the Pakistani community in Denmark was strongly affected by a huge earthquake in Pakistan in October 2005. Even though this disaster took place at a specific location, reactions to it came from all over the world. Mikkel Rytter, the author of the chapter, claims that this was largely due to the global media, which transformed it from a local to a worldwide event. The Danish-Pakistani community was strongly involved in the reactions. Many of them sent money, and a lot of the medical doctors in their midst even went to Pakistan. This gave them a great deal of symbolic capital ‘back home’ in Denmark, which they tried to exploit to improve the general image of Danish Muslims. These dynamics bring Rytter to the conclusion that, in the current era, shocking happenings can not only turn into huge events in the specific locations where they take place, but indeed all over the world.

This is a critique of what we could call the *eventful distance hypothesis*: the idea, which is quite prominent in social scientific theorizations about events (e.g. Berezin and Diez-Medrano, 2008: 8; Legewie, 2013: 1231–2), that the closer one gets to the place of the happening, the stronger its effects will be. The example of the Danish-Pakistani Muslims indicates that this is particularly a matter of *emotional* rather than *physical* distance (although there probably exists a strong correlation between the two). However, this also brings up the question of where the most interesting eventful dynamics actually take place. Is this necessarily always at or around the location of the happening? Based on my own research – a PhD thesis about meaning-making processes with respect to 9/11 within the American, French and Dutch public spheres – I would not draw this conclusion. It would certainly be interesting if a researcher had been in New York or Washington around September 11th, 2001, to see how people living in those two cities were grieving and trying to make sense of the happening. Yet in my research I also find that 9/11 has been a highly important event in the Netherlands. It has become one of the main signifiers for many Dutch people to believe that Islam is incompatible with ‘Western’ or ‘Dutch’ values (Van Dooremalen, under review). Accordingly, I think that it would also have been very informative to spend time in native Dutch communities during the first days after the attacks, to observe if and how their feelings about Muslims were changing.

Another question that occurred to me while reading the book is how fruitful a strict focus on ‘creative situations’ is for understanding events. Many of its contributions centre on one happening or even one moment. This is a refreshing contrast with overly structuralist accounts in which individual happenings almost seem to disappear from history. However, in my view it becomes problematic at the point where this approach implies that events are social universes *unto themselves*, which take shape more or less independently from larger societal structures. For example, Chapter 6 argues that one demonstration during the Danish Cartoon Controversy in 2005 caused the creation of one coherent Muslim identity in Denmark. It was the first time that Danish Muslims with various ethnic backgrounds came together and felt that they were part of the same collective. I do not want to underestimate the general feelings of anger about these cartoons within the Danish Muslim community or the solidarity that might have been a performative effect of the demonstration. Yet I think it goes too far to imply that these cartoons and the demonstration *in themselves* were enough to create this Danish Muslim identity. There must have existed a fertile ground for the emergence of these feelings of solidarity. Such feelings, or any type of event effects, do not arise a-contextually but are always created by the *interaction* between a significant happening and feelings and ideas that have been present for a while.

*The Political Aesthetics of Global Protest: The Arab Spring and Beyond* (2014) focuses far less explicitly on events than *In the Event*. This is a book about the various social movements that emerged during and in response to the happenings of the Arab Spring, such as protests against the dictatorial regime in Tunisia, anti-corruption activism in India and the various Occupy movements in the United States and Europe. The central thesis of the book is that these movements are united in their use of a ‘performative aesthetic’: images, songs, videos, and dramatic performances were part of the protests at Tahrir Square in Egypt as well as of the Greek anti-austerity movement that came about in 2011. As the editors argue in the introductory chapter (p. 2):

They [the protests] were not simply echoes of earlier protest movements, but utilized innovative political aesthetics in an age of global media and social networking; their material, visual, physical and sensual manifestations were a means of mobilising and contesting corruption, inequality, autocracy and neoliberal policies.

This first chapter is followed by 14 chapters, each covering another protest movement. They are written by different scholars, who are all specialists in their particular fieldwork sites. Like the contributors to *In the Event,* they did not go to these locations specifically to study the events, but were already working in these countries when the protests started. Yet even though participant observation plays an important role in substantial parts of the book*,* the chapters are written less from a personal, in-depth perspective than in *In the Event*. They are, instead, more essayistic.

There are no explicit claims in the book stating that it wants to add to the ethnographic study of events. However, there is an implicit idea that the ‘political aesthetics’ constituted an eventful, creative aspect of all these protest movements. For instance, in Chapter 3, Dahlia Wahdan shows how the songs that were invented during the occupation of Tahrir Square were not only used to *describe* the situation at hand but also to *make people move*. They were not ‘songs of revolt, but also songs that revolt’ (p. 55). She continues (p. 56): ‘they [the songs] seek to highlight, establish and perpetuate a condition of turbulence and rejection of any form of oppression. As such they emerge as media of revolution and change’.

I am rather ambivalent about this claim. Indeed, I can see how aesthetics have been important for the various protest movements. But is this really something new? Is it not an integral part of every social movement to use all sorts of aesthetic symbols in order to reach its goals? Think of how communism, one of the most successful movements of the 20th century, spread its ideology. Images (of Marx, Lenin, the hammer and sickle), songs (‘The Internationale’), as well as films (*October: Ten Days That Shook the World*, Sergei Eisenstein’s celebratory depiction of the October Revolution of 1917), were omnipresent in its propaganda campaigns. The same can be said about many other movements, such as the Nazis’ usage of the media to spread their ideology or the protest songs of the American 1960s’ civil rights movements. The book’s claim could potentially relate more to the *global spread* of this aesthetic, suggesting that what was really new was that a similar type of performative protest became diffused all over the world. Indeed, it has been argued that the possibility to unite a wide variety of activist groups with sometimes even conflicting political goals within the ‘brands’ of Anonymous and Occupy has been an important factor in the successful worldwide diffusion of these social movements (Beraldo, 2017). However, in that case, *The Political Aesthetics* should have focused more explicitly on the worldwide *distribution* of these aesthetics than on their usage per se.

What predominantly holds all of these forms of protest in the various countries together is that even though they were aiming to achieve different political goals, they all took shape within a period of less than three years. Could there be a reason for this quick succession? Is it possible that one event (the Arab Spring) inspired the other (Occupy)? The editors of *The Politics of Aesthetics* seem to assume that this is the case (as suggested by their inclusion of a timeline in which all the protest movements are juxtaposed). But since the chapters are all written independently from one another, they do not delve into this. Only the chapters on the Arab Spring (Chapters Two – Six) indicate the existence of this mechanism to a certain extent, by expounding on how the various protest movements in the different North African countries referred back to one another.

A question that then follows from event literature is: what made all of these movements so eventful if their performative aesthetics were not novel? Perhaps it was the very fact that they came about? By showing their disagreement with the *status quo* in their respective countries, the protesters indicated that the consensus in their societies was not as widespread as common sense thinking might have presumed. By raising their collective voice and coming together on streets and in squares, the protesters in North Africa made clear that a strong call for democracy existed in these countries, and Occupy showed that there is a lot of discontent with the current state of capitalism in many Western countries. The eventfulness of the movements was thus not so much related to their *styles of performing,* but to their *contents*.

Yet even though the book’s central empirical claim might be dubious, it offers a notable methodological contribution: it shows that – as long as the fieldwork consists of more or less the same empirical materials – the ethnographic method can be used to compare similar types of events in different countries. In the case of *The Political Aesthetics,* this has resulted in an interesting overview of how the same types of performativity were used by protest movements all over the world.

*The Occupiers* (2015) has a much more specific subject of study. It is mainly an examination of the Occupy movement in New York, although it also offers insights on how the same movement took shape in other American cities (Philadelphia, Chicago) and European countries (Spain, Greece). Like the other two books, it indicates that having the right intellectual and social capital are necessary conditions for producing an ethnography of events. However, while the authors of the various chapters of *In the Event* and *The Political Aesthetics* were already doing fieldwork at the sites or in the countries they wrote about, Michael Gould-Wartofsky, author of *The Occupiers*, came from a different position. He was a graduate student at New York University when he noticed that the seeds of the Occupy movement were growing. Since he had already been part of leftist protest movements in New York and was doing coursework about social movements and state-capitalism relationships, he decided to dedicate his fieldwork to this upcoming movement. This shows, besides studying ones that occur spontaneously during fieldwork, a second option for conducting an ethnography of events. Yet the challenge of this option is that one often has to be quick, since most big happenings do not last that long. A protest might last for weeks or months, but the attack on the Twin Towers or the fall of the Berlin Wall happened unexpectedly, and the biggest ruptures were probably taking place during the first few days immediately afterwards. Accordingly, in order to capture the happening’s most important meaning-making dynamics, one has to arrive on the fieldwork site rapidly, which is often difficult.

Gould-Wartofsky has made an ethnography of the Occupy movement that goes back to the moment it started, in September 2011. He has even included the anterior months, when leftist New Yorkers came together in meetings to discuss how they could set up a huge new form of protest against capitalism. Then, he tracked the genesis of the movement over the months that followed by doing participant observation among the Occupiers in New York, as well as through conducting in-depth interviews with 40 of them and another 40 in other cities. His perspective on the movement is that it fits with a larger pattern of protest movements such as those that were part of the Arab Spring and the ones that occupied squares in various European cities (e.g. the Indignados in Spain). Furthermore, Gould-Wartofsky also highlights the important role of the disappointment in the Obama presidency among many leftist Americans. Consequently, in contrast to various analyses of *In the event*, the happenings described in *The Occupiers* are not analysed as if they occurred in isolation but rather are clearly related to greater societal trends.

The occupiers thought carefully about their name and slogan (The 99 Percent), and they studied how prior movements had been occupying squares. This shows us an interesting *paradox of eventful surprise*. Most events are, to a large extent, planned. Not only those created by protest movements, but also terrorist attacks or genocides. Yet to the others (the non-protesters or the non-terrorists), these plans should be unknown. They have to be surprised by a happening, because otherwise it will not that easily turn into an event (Sewell, 2005; Wagner-Pacifici, 2017).

*The Occupiers* is largely ‘in the event’, to quote the title from this review’s first book. It gives a wonderful inside view into the meetings, feelings and ideas of the protesters. As this is its central aim, the book focuses less on the transformative capacities of the movement. However, towards the end of the book, Gould-Wartofsky quotes interviews with the Occupiers who claim that the impact of the movement has been to ‘change the conversation’ about state-capitalism relations. Subsequently, the book, published in 2015, proposes how the discontents that were part of the Occupy movement might lead to a manifestation of these feelings in the political arena (pp. 225–6):

both Democrats and Republicans will have to contend in the coming years with an emerging anti-corporate coalition of newly mobilized Millennials, organized labor, and disenfranchised constituencies of color. If the major parties fail to take on the crisis of inequality, they may face a proliferation of third-party challengers at the grassroots.

This is basically what we have seen with the presence of Sanders (and perhaps also Trump) as a contender for the presidency of the United States.

A problematic point regarding this question of impact is that the movement, or the event itself, is probably not the best indicator of long-lasting forms of transformation. For many protesters, participation in the Occupy movement must have been an important event in their individual lives. However, to find the major, societal impact, it would be more productive to focus on actors who were not directly involved in the event, but had the political or discursive power to do something with it on a larger scale. This could consist of analysing public actors in the media, following politicians in parliamentary debates and during elections, or reading reports of financial organizations. Although Gould-Wartofsky writes that many Occupiers have a distaste for such ‘elitist’ practices, this is ultimately where the big societal impact is going to be created. Another point of concern is the issue of when the event actually ends. Is the event *only* what happened during the first days, weeks or months of rupture? Or does it also include what goes on in reference to it in the years afterwards? In my PhD thesis, I take the second approach of not binding the event to time (cf. Wagner-Pacific, 2017), and I show that after the Charlie Hebdo assault in Paris in January 2015, the attacks on the Twin Towers were still seen as a motive for policy changes in each of the three countries I consider (Van Dooremalen, under review). However, using such an approach poses another problem to ethnographers, because it would be highly costly and time-consuming to continue to follow an event for such a long period. Indeed, anthropologically-oriented historians such as Zemon Davis (1975) and Le Roy Ladurie (1976) have come up with long-term micro-analyses of events. However, given their historiographical character, those investigations lack the first-hand experience and observation that make ethnographic research, such as that presented in *The Occupiers*, so powerful.

In sum, this analysis brings me to the following conclusion regarding the usage of ethnographies to study events. All three books show that the method is well-suited for capturing the shock and rupture that are part of people’s lives during eventful periods. *In the Event* demonstrates how distinct the actions of people in these periods can be from their behaviour in ‘settled times’ (Swidler, 1986). *The Political Aesthetics* gives an interesting overview of how in almost every part of the world, and in a period of only a few years, disruptive protest events came about. Finally, *The Occupiers* presents a wonderful inside account of the whole life-cycle of one protest event. The ethnographic method thus has quite a lot to offer for the study of events.

Nevertheless, using this method also brings about difficulties. First, there are practical issues, such as the possibility of being at the right place at the right moment and having the time and money to conduct research. These are all necessary conditions for researching an event, but hard to achieve. Second, as transformative responses to events are not bound by the place or time of the happening, other methods (interviews, discourse analysis, surveys) might be more suitable for getting a comprehensive picture of their meaning-making dynamics.
